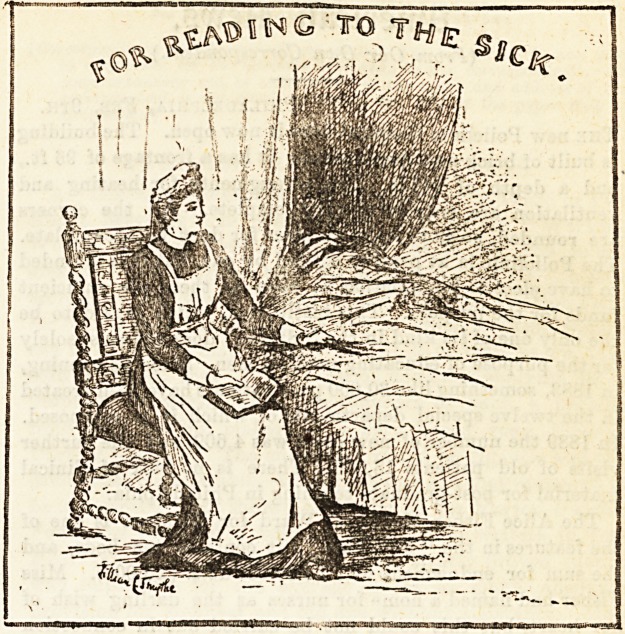# Extra Supplement—The Nursing Mirror

**Published:** 1891-03-07

**Authors:** 


### "The Hospital" Nursing Mirror.

Being the Extra Nursing Supplement of "The Hospital" Newspaper.

Contributions for this Supplement should be addressed to the Editor, The Hospital, 140, Strand, London, W.C., and should have the word "Nursing" plainly written in left-hand top corner of the envelope.

### En Passant.

WORCESTER CITY INSTITUTION.—The twelfth annual report of the Worcester City and County Institution of Trained Nurses says that 366 applications for nurses were received last year, and 256 were supplied. The staff now consists of 35 nurses and seven probationers. There is a small adverse balance, due to the influenza having incapacitated several of the nurses for months, and due to the fact that the committee have given four of the nurses midwifery training during the year. The institution has been affiliated with the Queen's Jubilee Institute, and the Lady Superintendent and nurses have received their badges. The report remarks that this is the second time the Lady Superintendent's work has received Royal recognition, for in 1880 she was presented by the Duchess of Edinburgh with a medal to commemorate her devotion and skill during the small-pox epidemic at Gravesend.

THE MIDDLESEX HOSPITAL NURSING INSTITUTE.—The Trained Nurses' Institute established some years ago in connection with the Middlesex Hospital, has been most successful during the year 1890. Over £1,000 has been transferred to the capital of the fund, this being made up of £384 profit on the nurses' earnings, and £726 excess of income over expenditure in respect to the Lady Prabationer Fee Fund. The report presented by the Secretary Superintendent (Mr. F. Clare Melhado) to the quarterly meeting held on the 26th ult., stated that "While still retaining five years as a minimum period of the training required to qualify a nurse for admission to the institute, a few exceptions have been made allowing nurses possessing superior qualifications to enter the institute after a training of three years. There are now nineteen nurses attached to the institute; their services are in constant demand, and everything goes to show that the enterprise is making good progress.

MISS NIGHTINGALE.—In General Sir Edward Hamley's book, "The War in the Crimea," which has just been published, full credit is given to Miss Nightingale for her marvellous power of management, as well as for her direct services to the sick. Speaking of the arrival of the little band of nurses at Scutari, the author says: "Thus began to enter into the history of the contest an element which strongly moved the imagination of the community, both from the extraordinary alleviation of suffering and establishment of order which it effected, and from the contrast which its gentle and beneficent character offered to the gloomy terror of the war." In another paragraph General Sir Edward Hamley says: "A regular correspondence was established between Miss Nightingale and Lord Raglan. Receiving such support, as well as that derived from the strong interest which the public evinced in her mission, she gradually acquired powerful controlling influence, and to this the extraordinary improvement in the condition of the hospital which ensued was then, and has continued to be, chiefly ascribed. The excellent medical staff cheerfully accepted her sway, and the skill and energy which they had always given without stint, no longer expended in struggling amidst chaos, were directed to the best ends." The whole book is the clearest and most interesting account of the Crimean War we have ever read.

A LADIES' DEPUTATION.—It was a capital notion of the Committee of the Midwives' Registration Bill to send a deputation of ladies to wait on Lord Cranbrook with reference to the subject. The deputation was well chosen also, representing all sections of women. Lady Aberdeen was at the head, and, in introducing the deputation, said that many of the leading members of the medical profession were in favour of the principle of the Bill, and it was only for the principle that the deputation desired to see the measure read a second time. Lady Lucy Hicks-Beach said she was told that the objection was made that registration of midwives would tend to interfere with medical practice. So far from the Bill prejudicing the doctors, it would tend to save their time by ensuring the employment of women competent to judge when the doctor ought to be called in. Mrs. Smith said that she had had twenty years of experience among poor women, and it was her opinion that nothing but compulsory registration would cure the evils which existed. Dr. Annie M'Call said they did not want the midwife to take the place of the doctor, but to act as his assistant, and that she could do with six months' training. As for the opposition of general practitioners, it must be remembered that no Bill of the kind ever passed without strong opposition. Miss Wilson said that the exception now was to find a midwife with any sort of certificate in the rural districts, and few of them were competent to form an opinion of a critical case. Mrs. Stuart Wortley, Miss Freeman, Mrs. Malleson, and Miss Rosalind Paget were also present. It is a pity women have not more weight in questions of this sort; anything more ridiculous than the fact that the Rev. Mr. Diggle is head of the section on "Infancy and Childhood" at the forthcoming Congress of Hygiene, it is impossible to imagine. Not a woman is down to speak on the subject.

PRESENTATION OF QUEEN'S BADGES.—On February 24th a meeting was held of the Manchester and Salford Sick Poor and Private Nursing Institution, the Mayor presiding. The Mayor said he hoped the largeness of the audience was an indication of the wide and deep interest taken in this institution. He would only say that in connection with the Salford branch there had been 2,000 cases, and that the nurses had made 42,000 visits; that in the Ancoats branch there had been 1,400 cases and 36,000 visits, and that in the Hulme branch—the most recently started—there had been 114 cases and 2,800 visits. The object of the present meeting was to distribute Queen's badges to the nurses who had qualified to receive them, and he would now call upon Mr. Oliver Heywood to do so. Mr. Heywood said the badges were conferred for training, experience, and high character in the service of a society affiliated with the Nurses' Institute, founded by the Queen in connection with her jubilee. Before a badge was given it was required that the nurse should have had one year of hospital training and six months of district training. The local nurses, however, had four years of hospital experience and three and a-half years of district training. Mr. Heywood then presented the badges. The following are the names of the superintendants and nurses who received them:—Ardwick and Ancoats Branch: Miss J. Blower, Lady Superintendent; Miss A. Evans, Miss S. Lake, Miss E. Healey, Miss M. Scott, Miss M. Holyoake, Miss M. Bennett, and Miss H. Davies. Salford Branch: Miss C. E. Barff, Lady Superintendent; Miss A. Simpson, Miss B. Kelly, Miss E. Ratcliffe, Miss C. Boddington, and Miss A. Marriott. Hulme Branch: Miss E. Hind, Lady Superintendent, and Miss S. A. Mills. The badges, which are to be worn on the arm, consist of the Royal monogram worked in pale blue silk on a ground of dark blue.

### Lectures on Surgical Ward Work and Nursing.

By Alexander Miles, M.B. (Edin.), C.M., F.R.C.S.E.

### Lecture XVI.—DANGERS OF CHLOROFORM AND THEIR TREATMENT.

(*a*) Difficulty in Respiration may be due to some foreign body getting into the larynx, such as false teeth, a piece of tobacco, or some vomited matter from the stomach; or it may be due to the paralysed tongue falling back, and so interfering with the entrance of air to the lungs. In the former case, the larynx should be explored with the finger, the patient's head being turned on one side to admit of anything falling into the cheek; in the latter the tongue should be seized with artery forceps and forcibly drawn out, thus opening up the glottis. The same result is obtained by turning the head on one side and pulling the chin forward, so that the lower teeth project in front of the upper ones. Sometimes the breathing is interfered with by the glottis closing through paralysis of the small muscles of the larynx itself. The symptoms of this condition are lividity and

Clover's Ether Inhaler.

a peculiar crowing, croup-like sound. Pull forward the chin, seize the tongue with forceps, and withdraw it, so as to open the glottis.

(*b*) Failure of the Heart, is usually due to reflex shock, the operation having been commenced before the patient was properly under; or to his having fainted from some other cause. To prevent this, give your patient some stimulant before commencing the administration, and don't hurry it. Never on any account place a patient who is partially under chloroform in the sitting posture. This is one of the most common causes of sudden death under chloroform. It is not death from chloroform, but rather from chloroformist. Should your patient faint, you must at once depress his head, holding him up by the heels if possible. Raising the foot of the table is usually sufficient. Give a hypodermic of ether or brandy; apply strong ammonia to his nostrils; flip him with wet towels; and, if at hand, apply the constant current. Never use an induced interrupted current which inhibits the heart by stimulating the vagus nerve.

(*c*) Vomiting is only dangerous in so far as it furnishes foreign bodies which are very apt to get into the air passages. The act, itself, also interferes to some extent with breathing. Never continue the administration if the patient has anything in his stomach to vomit, *i.e.*, if he be not prepared for chloroform. If he be prepared, on the other hand, and his stomach be empty, the sooner he is under the sooner the reflex act will cease.

Ether has long enjoyed what appears to have been a somewhat exaggerated reputation for safety. It is preferred by many on account of its stimulant action on the heart. It causes more struggling than chloroform, and in some patients the after-sickness and headache are more severe. In administering ether some form of inhaler is almost always used, as it is so very volatile that much is wasted with a towel. When given by the "open method," in which the ether is mixed with fresh air, Alli's inhaler is employed. This consists of a wire framework, on to which is threaded a length of flannel or domette bandage, and the whole is enclosed in an indiarubber case. By this method the patient takes a long time to go under, and struggles very much. By the "closed method," on the other hand, the anæsthesia is rapidly and quietly produced, by causing the patient repeatedly to breathe and rebreathe the same air charged with a gradually increasing proportion of ether vapour. This is effected by means of Ormsby's or Clover's inhaler. Remember that ether is a highly inflammable vapour, and must never be allowed near a light.

(3) "A.C.E. Mixture" consists of alcohol (absolute), one part; chloroform, two parts; ether, three parts. It has the combined advantages of ether and chloroform, the alcohol being added for pharmaceutical purposes. It should be freshly prepared, as the mixture decomposes readily.

Chloroform Drop Bottle consists of a small bottle furnished with a cork, through which two tubes pass, one reaching the fluid in the bottle, the other only piercing the cork and admitting air to take the place of the chloroform, which escapes by the longer one. It is used to save chloroform, and to enable the administrator to judge better of the amount he is using. The tubes are furnished with stoppers, which should always be replaced when the bottle is not in use to prevent evaporation.

Tongue Forceps.—Ordinary artery catch forceps are used to pull forward the tongue should it fall back and interfere with the respiration, or should there be any other cause interfering with the free entrance of air to the lungs. They should always be at hand, and perhaps the most convenient place to carry them is attached to the collar of the coat or apron strap. In seizing a patient's tongue take a good strong grip through the middle of the organ, and so avoid tearing pieces out of the edges, as invariably happens if you attempt to take a narrow hold.

The clean towel and solution tin are ready in case of sickness, and should be within reach of the chloroformist.

Hypodermic Syringes.—You should always have several of these on the table, and before the operation begins make sure that they are in working order. It is quite exceptional to find a hospital hypodermic syringe fit for use, and as there is almost no appliance which is wanted on such short notice, it is most important that it should be ready. A syringe may be wanted (1) to inject a solution of morphia and atropia before the operation, as this is supposed by some surgeons to render the administration of chloroform safer by diminishing sickness, preventing reflex stoppage of the heart, and diminishing pain. (2) To inject ether subcutaneously should the patient faint or the heart's action become feeble. For this purpose a syringe should always be filled with ether before the operation is begun, and placed near the administrator.

*To fill a hypodermic syringe*, remove the wire stillete from the needle, pour a quantity of ether into a minim glass, fill the barrel of the syringe without the needle, then screw on the needle, expel air from the apparatus, and place the syringe with the needle upwards, close beside a bottle of eucalyptus oil ready for use. Brandy and strong ammonia should always be at hand in case of emergency.

Cocaine is used as a weak anæsthetic, the stronger solutions—10 per cent. and 20 per cent.—being painted on to mucous surfaces, such as the nose and throat; while the 5 per cent. solution is injected hypodermically before performing minor operations of short duration. About 5 minims should be injected, and this repeated once or twice if necessary, the needle left *in situ*, and unscrewed each time to avoid unnecessary pain in re-inserting it. Its use is sometimes attended with dangerous symptoms, hence it must be employed with caution.

### Nursing Medals and Certificates.

THE LONDON HOSPITAL.

OUR largest training school for nurses gives no medal, but gives instead an extra certificate and prizes in money or books. When Miss Lückes became Matron of the London Hospital in 1880, she immediately set about organising theoretical and practical teaching for the nurses which was to occupy one year, and be followed by an examination. Sir Andrew Clarke delivered the first lecture to the probationers, and the Duke of Cambridge presented the first prizes won. The prizes are given annually to the three nurses who obtain the highest marks, and are to the value of five guineas, four guineas, and three guineas respectively. The Prize Certificate is ornamented with the above engraving, and is worded as follows: "London Hospital Nursing Home. Nursing Lectures and Examination, 18— to 18—. Prize Certificate Presented for Proficiency." It is signed by the Matron, House Governor, and Chairman. Two years ago honorary certificates were also instituted, to be awarded to those who nearly obtained the prize, and were above the average. The ordinary certificate of the London Hospital runs as follows: "This is to certify that............was received as a probationer on..........., and has completed her full term of two years' training in the medical and surgical wards of this hospital, both on day and night duty. During this time her work has been........., and her conduct has been........." This is signed by the Chairman, House Governor, and Matron, and is given to every nurse who serves her two years. But if the nurse has also passed her examination, the following is added: "........... has attended lectures on Elementary Physiology and Medical Nursing, and passed a.........examination. (Signed by the physician.) Also lectures on Elementary Anatomy and Surgical Nursing, and passed a.........examination. (Signed by the surgecn.)" At the bottom of the certificate is a little note stating that further particulars concerning the holder of the certificate can be obtained at any time on application to the Matron.

### DUE TO ALCOHOL.

ACCORDING to Dr. Richardson, the diseases attributable to alcohol are equivalent to one-tenth of all the diseases in the kingdom; no one will therefore be surprised to hear that the rate of mortality is higher from this cause than from pulmonary consumption.

FORSAKEN.

IT is an awful feeling that of being left to oneself without hope of help, whether it be expressed in the bitter cry of the child who thinks itself forsaken by it's mother, or the despairing "lost! lost!," of the man alone in a trackless forest, or drifting in an open boat on the vast ocean. But there is a still worse loneliness when the repentant soul, seeing clearly its sin and guilt, fears its Saviour's love has been too deeply outraged, and that it can never see His face again. Then it is we feel how helpless we are. Then we cry in agony, "My God, my God, why hast Thou forsaken me?" It is well for us that we should know how dependent we are upon our heavenly Father's care, but there is no necessity for despair. His love is so great that He is always ready to pour it out on us if we come to Him, but we are so self-sufficient, so presumptuous that we require a check to recall our better nature. We have, perhaps, been going on so prosperously and well that we have rejoiced in our success in life, and even said "I shall never be removed. Thou Lord of Thy goodness hast made my will so strong." Then God turns away His face, and we are troubled. We read of King Hezekiah that when the ambassadors of the princes of Babylon came to inquire of the wonders that were done in Jerusalem that "God left him, to try him, that He might know all that was in his heart." Alas! his heart was not given wholly to God; it was full of pride and vain glory at the riches and power which he displayed before the strangers, and though he afterwards repented and escaped the personal punishment of his sin, yet he brought trouble and misery on his country in after years. How necessary it is to remember whose we are, and whom we serve, and to pray constantly "leave us not, neither forsake us, oh! Lord God of our help." Without God we are weak and unstable; with Him we can do all things through Christ, who strengtheneth us. We gain courage from the knowledge that there is One always watching over us, to whom darkness and light are both alike. Never alone! oh! happy thought. Never left to fight our own battles against sin and wickedness; but with us is One who will comfort us on every side, who is about our bed, and about our path, who giveth His angels charge over us to keep us in all our ways, and who holdeth and preserveth us in the everlasting arms.

### American News.

(*From Our Own Correspondent.*)

Philadelphia, Feb. 9th.

The new Policlinic Hospital here is now open. The building is built of brick and terra-cotta; it has a frontage of 96 ft., and a depth of 143 ft. The arrangments for heating and ventilation are considered very complete. All the corners are rounded, so as to leave no place for dust to accumulate. The Policlinic is at present lighted by gas, but it is intended to have electric illumination as soon as there are sufficient funds for the purpose. This institution, which is said to be the only one of its kind in the "States," does not exist solely for the purpose of educating medical men. Since its opening, in 1883, something like 30,000 poor patients have been treated in the twelve special departments of which it is composed. In 1889 the number of new cases was 4,609, and the further visits of old patients 18,000. There is no lack of clinical material for post-graduate teaching in Philadelphia.

The Alice Fisher Memorial Ward for Children is one of the features in the "Policlinic"; it contains ten beds, and the sum for endowment will be 30,000 dols. (£6,000). Miss Fisher had named a home for nurses as the darling wish of her heart, but this could not be carried out in connection with "Blockley," a politically-managed hospital; so the memorial has been arranged by a few of her near friends to take a more practical form.

Miss Outlaw, who has just resigned her position of Superintendent of the Children's Asylum at Blockley, to become Head Nurse at the Policlinic, is one of the few nurses remaining at Blockley, who were trained by Miss Fisher. Undoubtedly, she gave up her life to the rescue of a politically-managed institution, and to its conversion into a good training school for nurses, as well as its proper equipment as a hospital with qualified nurses, instead of the careless and incompetent attendants of the former régime.

Thus Alice Fisher was a pioneer here, not in nursing especially, but in showing how refined womanhood could make its way through the complications of "politics in charge" of a hospital, and how attractive the social gifts of a woman could make the once dingy parlour of the pauper's home.

She ought to be remembered gratefully, and her memorial is properly put in a hospital ward. This is the best monument to her professional services, and none of those who loved and honoured Alice Fisher can for one moment dissociate her from the profession she served loyally and well.

The first annual meeting of the Nurses' Beneficial Association of Philadelphia was held in the Hall of the College of physicians, Dr. Wharton Linkler, in the chair. The Treasurer reported a balance in hand of 500 dols. (£100), one member had died, and six had received sick benefit. A sum of 700 dols. (£120) had been collected by the Committee to endow a bed in the "Policlinic," for the sole use of the members of the Association. The sum required by the hospital was 3,000 dols. (£600), but the room was now available, and a sum of 120 dols. (£24) had been spent by the Committee in furnishing the room.

Since the above meeting was held, a nurse, who is a member of the Committee, has received from her patient, Mrs. Harry Ingersoll, a sum of 3,000 dollars to be applied to the endowment of the above-mentioned bed in the "Policlinic."

The New England Hospital for Women and Children at Boston deserves notice; it is entirely officered by women. The Matron is Miss Gertrude Montford, a graduate of Bellevue, and at one time a worker in Rome; she has twenty nurses under her. Last year nine of the probationers graduated and received their certificates. The Nurses' Home caught fire last year and much damage was done, but the public subscribed 257 dols. to make good the loss. Dr. Charles Green lately addressed the nurses of the Boston City Hospital, and gave them the usual good advice about kindness and courtesy and the holding sacred of all learnt within the sick-room. The Bellevue School, New York, has a rule that a nurse is "to make herself generally useful." The Alumnæ members have held a meeting to protest against this rule, as in practice it leads to the family making a charwoman of the nurse, and depriving her of such rest as the case permits.

### A Winter's Holiday.

### V.—A SPANISH TOWN.

In due time we arrived safely at Laragoza, an essentially Spanish town, showing few traces of French influence. We secured a guide for the next day, and then went to bed. The guide appeared in good time, and proved most satisfactory. He really took charge of us for the whole day, and for a moderate fee he saved us from all persecutions of professional beggars and others, and he was very helpful in bargaining for the little purchases we wished to make. He took us first to the old cathedral, which was very grand, with its pillars of polished marble, its graceful architecture, and rich colouring. Truly a noble and beautiful edifice, in which the smallest bit of carving merited admiration. A great many people were praying in different parts, and there seemed to be several services in progress. The groups of worshippers formed fine pictures in themselves, and there was altogether so much to attract our notice that we should never have reached the next sight had not our guide remorselessly hurried us on. He next pointed out the walls of the Archbishop's palace, which looked very ancient, and so did the bridge across the Ebro, besides many of the buildings; some were in ruins and the others had been visibly "patched up." In the Leaning Tower we noticed that he took a kind of personal interest and pride.

After admiring it we proceeded to the other cathedral, dedicated to "The Virgin of the Pillar," which is a Moorish-looking and rather tawdry building. The painted wooden columns are of a graceful design and the roof is finely coloured.

The contrast between the two cathedrals was very striking —in the first we found fine oak, marble, and stone; in the second deal, plaster, and painted bricks. Behind the high alter in the newer edifice is placed a stone which is said to be part of the pillar on which the Virgin appeared, and as we stood near we saw one picturesque figure after another come rapidly up and reverently kiss first the stone and then the block on which it rested. They believe that all prayers breathed on this spot will assuredly be answered. Over one altar we saw a number of wax models of hands, feet, legs, etc., offered by diseased persons hoping for a cure. It was a grotesque collection of objects in truth! When the things increased beyond certain limits the superfluity was melted down into candles. We went into the sacristy to see a quantity of beautiful jewellery and many other treasures, all gifts which would be sold for the benefit of the church repairs, etc. Here also was exhibited the far-famed collection of mantles worn by the image of the Virgin at festivals—certainly they are miracles of costliness and triumphs of beauty and art.

Thence we returned to the streets and the sunshine, saw the market and admired the natural pictures made by the groups of people on the pavements, always changing, but always artistic.

Although the hot sun poured down on us the wind at the street corners was most piercing, and we understood why no Spaniard walked abroad without a shawl hanging loosely over his shoulders, ready to be drawn closely round throat and head, and leaving only the eyes to face the sudden and bitter blasts.

When we returned to the carriage we soon found ourselves passing from the town to the open country, and ascending an eminence we halted to observe the prospect, and here the heavy lumbering carriage was literally shaken by the wind. We had some difficulty in keeping our seats. Frances nearly lost her umbrella, and my bonnet threatened to disappear altogether. The guide told us that this was one of the posts held by the French army, and it certainly commanded an extensive view of the immense plain, stretching away beyond the large picturesque town. Vineyards were interspersed with tracts of uncultivated land, and everything was of an uniform hue. A "dead level" of monotonous colouring. Grey? Not exactly; an ugly slate colour, a cold neutral tint; no variation anywhere.

In a moment we understood why Spanish colours are so fine - even the poorest peasant exhibits a gay kerchief of rich hues. This brilliancy becomes a necessity which is begotten of the niggardliness of nature in clothing the land so sadly.

Just as we decided that we could not stand the wind and dust much longer, our guide gave the word for departure, and we were soon in front of the great circular building dedicated to bull fights. We found that we were expected to alight here, and after walking across the vast arena we inspected the smaller buildings in which the unfortunate beasts are confined whilst awaiting their turn. These places are so strong and so well guarded that it did not need the guide's words to assure us of the power and the mad fury of the poor animals who are trained and tortured for this sport. We declined to hear any more details, and gladly turned away from the weapons and instruments which appertained to the ghastly show.

On driving away we passed near a very large building, and on inquiry, were told that it was half occupied by "poor imbeciles," and the other half was a hospital for the sick: "Nothing to interest the signoras!" We instantly declared that we certainly must see what would interest us greatly. At first the man evidently thought that we did not understand what we were talking about, and he repeated his explanation. At last, finding that we were in earnest, he condescended to obtain leave from the porter for us to enter, but did not disguise his surprise at this extraordinary fancy in ladies who had not seen, and evidently had no wish to see, a Spanish bull fight! He led the way into a corridor, and thence up a marble staircase to the women's wards. Over each door we noticed that a name was placed, and we peeped into a "pay ward," which was very dainty, with blue satin quilts edged with lace on the beds. The whole place seemed bright, and sweet, and clean; we were not fortunate enough to see any of the presiding nurses, and we soon retreated, bearing with us a pleasant impression of the only Spanish hospital we happened to meet with.

### Princess Christian's Daughter.

Miss E. Durham, Farringford, Freshwater, Isle of Wight, acknowledges the following additional subscriptions towards a wedding present for Princess Louise of Schleswig-Holstein to be given as a proof of the gratitude of nurses for the interest Princess Christian has ever taken in their progress. Subscriptions will be received until the end of March, and will be acknowledged in these pages. From February 23rd to March 2nd the following sums were received:—

Superintendent M. Going, 5s.; Matron E. G., 2s.; Army Nursing Sister, 2s. 6d.; Sister C. Waddington, 2s. 6d.; Nurse Shoosmith, 2s. 6d.; Nurse Edwards, 2s.; E. Sloggett, 2s.; Ada Thompson, 2s.; A. Webster, 2s. *Nurses* (1s. each): E. Still, K. Stannett, C. A. Richards, Edith Allsop, E. Gillingham, A. Garrett, S. M. Hayes, E. Parker, E. M. Burgess, Caroline A. Bone, Georgina Redit, Nurse Sylvia, Nurse Hettie, Nurse Watson, Nurse Lowe, Nurse Oxtoley, and Nurse F. Green.

Nurses Robertson and Rouch are informed that their subscriptions of 2s. 6d. each were received and acknowledged.

### Everybody's Opinion.

*[Correspondence on all subjects is invited, but we cannot in any way be responsible for the opinions expressed by our correspondents. No communications can be entertained if the name and address of the correspondent is not given, or unless one side of the paper only be written on.]*

### A PENSION FUND FOR SERVANTS.

A "District Superintendent" writes: "A Word or Two About Servants" in your article, No. 19, on "Working Women in Large Towns," emboldens me to ask you to call attention to the urgent need for a pension fund for domestic servants. The grand success of the National Pension Fund for Nurses surely indicates one way in which working women can be helped to help themselves. If it be true, as we have seen it stated, that 70 per cent. of domestic servants die in hospitals or workhouses, the founders of such a pension fund will be the makers of a very broad road through Darkest England. The enterprise would be gigantic and the difficulties innumerable—till three years ago they appeared to be insuperable—but the establishment of the Nurses' Pension Fund has removed a host of them. The writers in The Hospital, in advocating their fund, clearly proved that it was impossible for the average nurse to make adequate provision for sickness and old age without assistance. How much more difficult is it for the average domestic servant? Let those who doubt the difficulty make the calculation how long it would take the servants of their own household—supposing they never were ill or out of situation and never gave a penny to their relatives—to lay by in the Savings Bank as much as would yield them even a shilling a-day in their old age. To look at it from another point of view: What would tend more to increase the supply of good servants than such a pension fund, or be more certain to bind together by ties of mutual respect and affection mistress and maid, master and man?

### Appointments.

[It is requested that successful candidates will send a copy of their applications and testimonials, with date of election, to The Editor, The Lodge, Porchester Square, W.]

Bristol General Hospital.—Miss Mary Eaton has been appointed Matron of this hospital. She was trained at the Derbyshire General Infirmary, and had charge of wards for five years. Afterwards she was Sister of the Royal Albert Hospital, Devonport, for eight months. For the last nine months she has been Night Superintendent at Bristol.

London Hospital.—Miss Morgan has been appointed Sister Blizard, and Miss Louisa Herrman has been appointed Sister Queen: both trained at the London.

Leavesden Asylum.—Miss Craig, Matron of the City of Birmingham Hospital, has been appointed Matron of the Leavesden Asylum, under the Metropolitan Asylums Board.

Paignton Cottage Hospital.—Miss Annie Cheatle has been appointed Matron of this new hospital. Miss Cheatle trained at the Torbay Hospital, and remained there some time; she has also had experience at the Victoria Park Chest Hospital.

Southampton Eye and Ear Hospital.—Miss Clara Jaggars has been appointed Matron of this new hospital. She trained at the Radcliffe, and has worked at Blackheath and in Ayr County Hospital.

Western Dispensary, Rochester Row, Westminster.—Miss M. Whalley, diplomée of the London Obstetrical Society, has been appointed midwife to this dispensary, and also outdoor midwife to the Westminster district of the General Lying-in Hospital, York Road, Lambeth.

### Nurse Hilary.

(*Concluded from page* cxxiv.)

ALLAN and Sister Hilary walked away to a seat under a lilac tree in the hospital garden, and there she told him all. "I did not know; I thought she was your wife," she cried. "I have no wife," said Alan, "*yet.*"

He took her hand in his.

"She is my cousin Alan Webster's wife. Theirs is a sad story. She ran away with him when she was very young, and he a ne'er-do-well sort of fellow. They were in desperate straits when I came across them in London, and helped them a bit now and then"—his honest face reddened—"until I was in such precious low water myself. And when I went away thinking you had taken me at my word and chosen the path of wisdom"—here they both smiled—"I found that he had enlisted at the last minute in the same regiment. He is twice the fellow he was—service has brought his grit to the fore; and what 'on duty' means you know."

He looked at her proudly, and then there was a long, happy silence. Mrs. Webster and her husband were sitting together when they appeared again. She looked up smiling through happy tears; and he, a slight, dark, young fellow, gave Sister Hilary a hearty handshake.

"You have not changed much, Alan," said Mrs. Webster, "since the photo you gave me before you went away."

"And here is yours," said Alan, giving it to Hilary. "It has travelled far and wide and been in many strange places, but it has found its owner at last, though *not* in the Green Park."

Mrs. Webster's husband touched the Victoria Cross that shone on Alan's coat.

"If it had not been for you, old fellow, I should not have been here," he said, brokenly.

A stout, elderly figure, shining in brown silk and many bugles, a wild and flowery bonnet nodding above her cheerful face, came trotting towards them.

Sister Hilary went to meet her, and the rest looked curiously round.

"Mother! Mother!"

The wild cry burst through the still garden.

Mrs. Webster had risen to her feet. Lady Beckett's arms were round her.

"Eleanor, my little girl, at last, at last!"

"It ought to be here," said Alan, as Sister Hilary laid her hand lovingly on the Victoria cross. "Here," touching the bib of her snow-white apron.

"Oh, nurses can win medals, too," answered Hilary, her face as bright as the May sunlight above them, "but, after all, you have given me the reward a woman wants the most."

"And what is that?" said Alan.

"Love," said Sister Hilary.

"As a cross or a crown?" asked Alan, mischievously.

"*Both,*" said Sister Hilary, stoutly. A. V. M.

---

THE next meeting of the Hospitals Association will be held on March 18th, at eight o'clock, when the subject for discussion will be the Midwives' Registration Bill. Tickets can be procured from the Hospitals Association, 140, Strand.

### "There Was a Time."

There was a time, a dreadful time,
When I was but a Pro.',
A hapless, most unlucky one,
Who nothing right could do.

A time when I the lotions spilt,
And smashed the dressing dishes,
And spent my brief "free time" in nought
But vain regrets and wishes.

A time I thought I'd never know
What next the doctor wanted.
With visions of most wild mistakes
At night my dreams were haunted.

A time when bandages and splints
Were hopelessly entangled,
And all my mind was taken up
With ways and means new-fangled.

A time when probe and spatula,
Iodoform and plaister,
Conveyed but one idea to me—
The dread of some disaster.

A time when "dressings" were as Greek,
And when I'd not a notion
Why sometimes they carbolic used
And not boracic lotion.

There was a time when first I stood
And watched an operation,
And thought that nursing never was
Nor could be my vocation.

A time when I was always wrong
And always lived in terror,
Lest I should cause a patient's death
Through some most fatal error.

A time when all the patients seemed
Resolved to fret and worry,
And wanted fifty things at once
When I was in a hurry.

A time when every day I had
To rise at six precisely,
Just when I thought I'd settled off
To go to sleep so nicely.

A time when I had to submit
To constant supervision,
And learned to carry orders out
With promptness and decision.

A time when I had not the chance
To grow the least conceited,
So often were my best attempts
Most signally defeated.

A time when all the praise I got
Was, "Well, you might do better!"
Although I thought I had obeyed
The order to the letter.

There'll come a time—a happy time,
When all my training over,
I'll be a full-fledged "Sister" then,
And so I'll be in clover.

A time when I no more shall be
So slow of comprehension,
And when to be my friend will not
Be quite a condescension.

A time when I shall get beyond
My weary year's probation,
And in the sweetest frame of mind
Complete my education.

A time when I will always be
To new pro.'s most forbearing,
And of my public reprimands
Will ever be most sparing.

A time when I shall ne'er forget
My first twelve months' hard labour,
And ever merciful will be
To my probation neighbour.

E. M. F.

---

### Amusements and Relaxation.

N.B.—Word dissections must be sent in WEEKLY not later than the first post on Thursday to the *Prize Editor*, 140, Strand, W.C., arranged alphabetically, with correct total affixed.

The word for dissection for this, the TENTH week of the quarter, being "IVANHOE."

| Names. | Feb. 26th. | Totals. | Names. | Feb. 26th. | Totals. |
|---|---|---|---|---|---|
| Reynard | — | 77 | Woodbine | — | 25 |
| Reldas | 21 | 414 | Madame B. | — | 25 |
| Tinie | — | 30 | Shakespeare | — | 59 |
| Patience | — | 76 | Smyrna | — | |
| Jenny Wren | 22 | 368 | Southwood | — | 102 |
| Agamemnon | 24 | 407 | Gipsy Queen | — | 21 |
| Wyameris | 22 | 391 | Snowball | — | 19 |
| E. C. | 22 | 406 | Rita | — | 88 |
| Ecila | — | | Mortal | — | 16 |
| Hope | 24 | 401 | Nurse Annie | — | 15 |
| M. W. | 22 | 402 | Carmen | — | 11 |
| Qu'appelle | 19 | 485 | Grannie | — | 45 |
| Nil Desperandum | 23 | 409 | Amie | — | 30 |
| Lady Betty | 21 | 385 | M. R. | — | 25 |
| N. A. S. | 17 | 331 | Primrose | — | 24 |
| Sister Jack | — | 62 | Nurse J. S. | 81 | 199 |
| Crystal | — | 203 | B. A. C. | 22 | 124 |
| | | | Theta | 21 | 21 |

---

### Notes and Queries.

### Queries.

(38) *Feeding Babies.*—I have heard the theory advanced by medical men, that babies should not receive nourishment between eleven p.m. and seven a.m. Has any nurse put this theory into practice, and with what result?—*Nurse.*

(39) *Nurses' Hours.*—Mr. Reade, of Charing Cross, said before the Lords' Committee, that the nurses were on duty only ten hours. Is this the only hospital so managed, or can your readers tell me of others?—*E.*

(40) What London or county hospitals admit on their staff nurses not of their own training?—*E.M.S.*

(41) Where can I get a pattern of the Sister Dora cap?—*Edith.*

(42) Wanted the names and addresses and the best way of getting a little girl with hip disease into an incurable home.—*Incurable.*

(43) Has any nurse seen burns treated with grated raw unwashed potatoe? If so, with what result?—*Pomme de-terre.*

### Answers.

*Dilemma.*—You are eligible to try for the prize again, and we hope you will do so and will be successful.

*Nurse E. Payne.*—Your paper was received too late for competition; it was correct.

*Nurse Lucy.*—You can only receive the badge of the Jubilee Institute if the society for which you work affiliates with the institute.

*T. H. B.*—It is a standing rule, to which we can make no exception, that the names of particular practitioners are never given. You should consult your own medical man about any specialist you may desire to see.

## Figures and Tables

**Figure f1:**
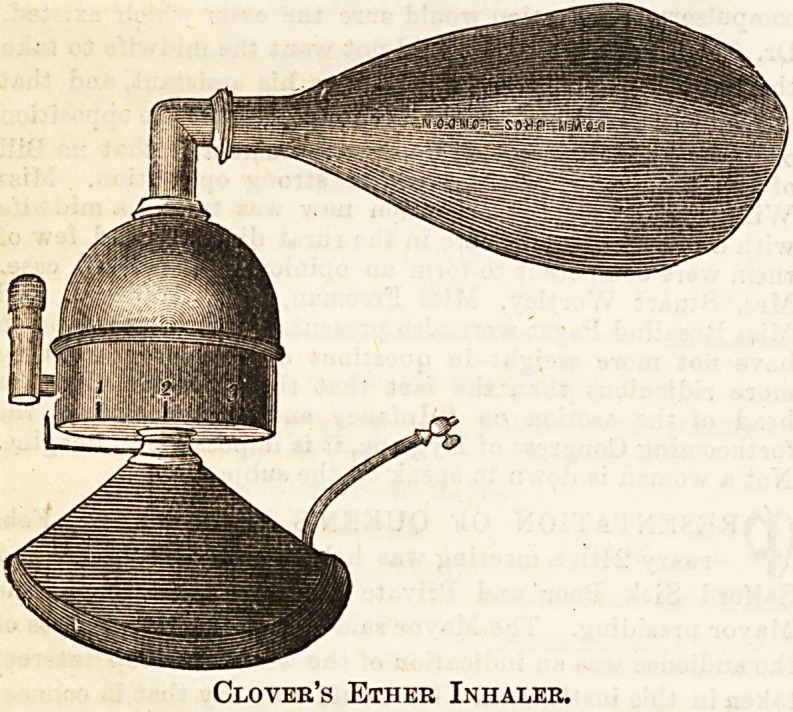


**Figure f2:**
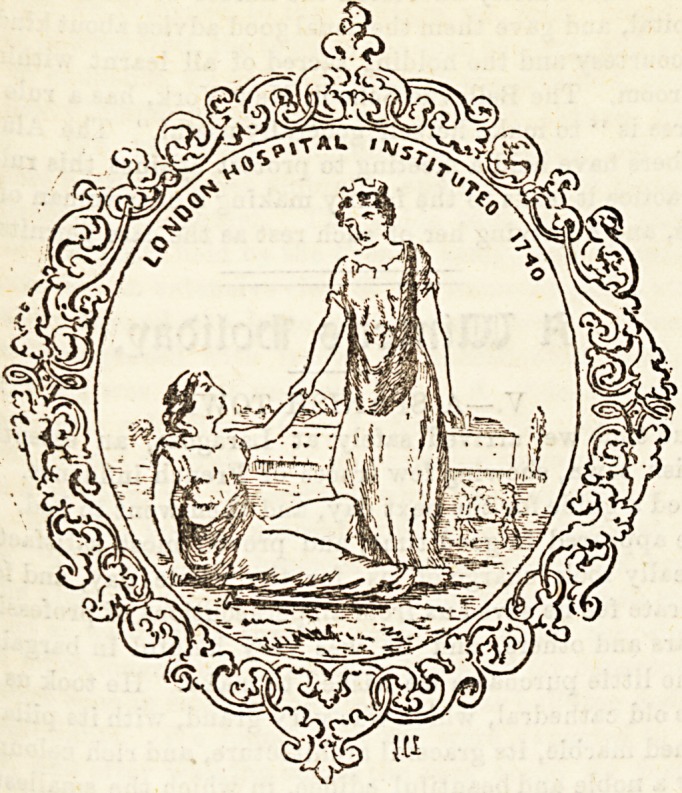


**Figure f3:**